# AUSPEX: An integrated open-source decision-making framework for UAVs in rescue missions

**DOI:** 10.3389/frobt.2025.1583479

**Published:** 2025-08-12

**Authors:** Björn Döschl, Kai Sommer, Jane Jean Kiam

**Affiliations:** Department of Aerospace Engineering, Institute of Flight Systems, University of the Bundeswehr Munich, Neubiberg, Germany

**Keywords:** AUSPEX, UAV, decision-making, planning, search and rescue, holistic framework

## Abstract

Unmanned aerial vehicles (UAVs) have become paramount for search and rescue (SAR) missions due to their ability to access hazardous and challenging environments and to rapidly provide cost-effective aerial situational awareness. Nevertheless, current UAV systems are designed for specific tasks, often focusing on benchmarking use cases. Therefore, they offer limited adaptability for the diverse decision-making demands of SAR missions. Furthermore, commercially available integrated UAV systems are non-open-source, preventing further extension with state-of-the-art decision-making algorithms. In this paper, we introduce Automated Unmanned Aerial Swarm System for Planning and EXecution (AUSPEX), which is a holistic, modular, and open-source framework tailored specifically for enhancing the decision-making capabilities of UAV systems. AUSPEX integrates diverse capabilities for knowledge representation, perception, planning, and execution with state-of-the-art decision-making algorithms. Additionally, AUSPEX considers the heterogeneity of available UAV platforms and offers the possibility of including off-the-shelf and generic UAVs, with an open architecture into the AUSPEX ecosystem. The framework relies only on open-source components to ensure transparency, as well as system scalability and extensibility. We demonstrate AUSPEX’s integration with the Unreal Engine-based simulation framework REAP for software-in-the-loop validation and a platform-independent graphical user interface (AUGUR). We demonstrate how AUSPEX can be used for generic scenarios in SAR missions while highlighting its potential for future extensibility.

## 1 Introduction

### 1.1 Motivation

Unmanned aerial vehicles (UAVs) are increasingly used to improve mission efficiency in domains, including logistics, surveillance, environmental monitoring, and rescue operations. Their ability to quickly access otherwise impervious areas and lower operational costs compared to conventional methods make them particularly valuable in time-sensitive emergencies (Lyu et al., 2023).

However, the highly diverse conditions pertaining to search and rescue (SAR) missions require UAVs to operate in only partially observable, complex environments while adhering to strict time constraints, minimising risks for both rescuers and victims. Their deployment spans various environments (Vincent-Lambert et al., 2023), from man-made to natural disasters and from maritime to mountainous areas. In all cases, flexibility to adapt to changing mission scenarios is a critical factor (Hayat et al., 2017; Vincent-Lambert et al., 2023).

Increasing UAV fleet sizes is promising but presents multiple challenges, including a higher personnel demand in rescue services, which may not be possible or may also result in decreased cost-effectiveness^1^. Furthermore, as UAVs may be acquired at different times in practice, the UAV fleet is likely to be heterogeneous. As a result, interoperability between different UAV units cannot be guaranteed.

Moreover, high-level automation in UAV systems presents enormous challenges due to the complexity of autonomous decision-making, coordination, and adaptability in dynamic environments (Alqudsi and Makaraci, 2025). Recent approaches address the various aspects of multi-UAV autonomy. Ribeiro and Basiri (2024) focuses on rapidly exploring unknown 3D environments using UAVs equipped with LiDAR sensors but struggled with coordination issues as fleet size increases. LEVIOSA (Aikins et al., 2024) achieves coordination of large-scale UAV swarms via natural language interfaces and reinforcement learning but is limited to static environments as it lacks the ability to handle dynamics arising in the real-time plan execution. The RACER (Zhou B. et al., 2023) framework uses a decentralised multi-UAV system, which is robust to communication loss, whereas Westheider et al. (2023) applied deep reinforcement learning for adaptive terrain monitoring. Although these approaches contribute valuable capabilities, these frameworks are highly customised for a single class of capability and often neglect the need for more holistic intelligence to enable UAVs to act in real-world complex missions.

Some recent efforts intend to address this shortcoming by proposing more holistic integration frameworks to include different capabilities, from planning to human–system interaction. Such frameworks are more usable and can be made to adhere to first responders’ standard operating procedures^2^. Commercial platforms like Auterion^3^ and Hivemind^4^ offer end-to-end solutions but impose hardware and software lock-ins, affecting future extension of hardware (e.g., UAV platform, payload and mission command, and control and communication) and software features (e.g., object recognition and mission planning). This can be a significant drawback as technologies in robotics and artificial intelligence (AI) are growing rapidly and proprietary systems deter the extensibility of mission capabilities. Other frameworks such as CERLAB-UAV-Autonomy (Xu et al., 2023) for simultaneous localization and mapping (SLAM) and MRTA Execution Architecture (Calvo et al., 2022) for multi-robot mission planning and execution, although open-source, hardly meet the requirements for flexible software and hardware extensibility, mainly due to tight architecture coupling and outdated dependencies such as ROS1^5^. Aerostack2 (Fernandez-Cortizas et al., 2024) takes a more modular approach and shows promise in lower-level controls, such as motion planning and computer vision techniques. However, it lacks components for high-level planning and execution and has not yet been used for realistic real-world simulation^6^, which can result in a lack of transferability from software to hardware development.

### 1.2 Contributions

In this work, we propose AUSPEX—Automated Unmanned Aerial Swarm System for Planning and EXecution—a holistic multi-UAV software framework designed to integrate UAVs into a decision-making support system, developed by considering typical requirements stemming from SAR operations. The advantages of AUSPEX are multifold:

#### 1.2.1 Modular architecture for scalability and flexibility

AUSPEX is designed and implemented in a modular manner, enabling *scalability in fleet size* ^7^ and ensuring *increased flexibility in* deployment, as the integration of a heterogeneous UAV into the system can be done independently from other existing UAVs in the system.

#### 1.2.2 Separation of high- and low-level autonomy

To leverage the advancement of modern AI techniques for an easier coordination of multiple UAVs, AUSPEX separates hardware-dependent low-level automation from mission-dependent high-level automation.

#### 1.2.3 Reusability and openness

AUSPEX is fully open-source, ensuring its reusability either as a whole by extending the software stack or partially by extracting only selected subsystems. Moreover, for real-world deployments, it is designed to support flight controllers with an open interface, leveraging their built-in fail-safe mechanisms.

#### 1.2.4 Human-in-the-loop and user interface

AUSPEX considers the first responders by providing a multimodal graphical user interface that enables human-in-the-loop operations.

#### 1.2.5 Realistic simulation-driven development

To accelerate the development from the laboratory to the open world without compromising safety, AUSPEX integrates a high-fidelity simulation environment capable of mirroring reality (i.e., operational environment and actual UAV platforms) so that validation of AI methods in decision-making can be performed extensively with software-in-the-loop validation before being deployed in the real world.

#### 1.2.6 Robust infrastructure with ROS 2

By adopting ROS 2 as middleware and communication backbone, it is possible to deploy AUSPEX as a *distributed system* while also improving real-time performance and scalability.

## 2 Related work

Robotics is increasingly important in SAR operations, from ground to amphibious and from marine robots to UAVs (Balta et al., 2017; Siddiquee and Ozkan-Aydin, 2024). Given the capabilities of UAVs to provide an “eye in the sky,” the deployment of UAV platforms in SAR operations has been a very popular choice. Survey papers by Lyu et al. (2023) and Vincent-Lambert et al. (2023) offer an overarching view of how UAV equipped with various sensors, including thermal imaging and LiDAR, are leveraged to increase efficiency in locating victims and risk mitigation in remote and hazardous areas.

For even more efficiency without escalating operational burden, multi-UAV systems with inter-platform operability and more substantial automation are necessary. This section provides insights into other initiatives focused on developing integrated multi-UAV systems, including mission controls for UAV fleets, as well as on AI methodologies applicable to coordinating single or multiple UAVs, to facilitate the extensive use of UAVs in SAR operations without increasing the size of the operating crew.

### 2.1 Integrated multi-UAV systems

Various multi-UAV frameworks have emerged, spanning both commercial and open-source frameworks. Auterion^8^ and Hivemind^9^ offer a robust end-to-end solution for UAV-fleet coordination, with partial modular software stacks, but both rely on proprietary components. Auterion depends on its own proprietary flight control unit to include new UAVs into the Auterion ecosystem, and Hivemind does not provide an ecosystem for integrating heterogeneous UAV platforms. As both systems consist of proprietary parts, software and hardware lock-ins deter the quick extension of the system to include additional UAV platforms or new high-level skills adapted to different missions. Open-source alternatives like the CERLAB-UAV-Autonomy framework (Xu et al., 2023) and the MRTA Execution Architecture (Calvo et al., 2022) provide single-to-multi-robot coordination frameworks with baseline SLAM, object detection, and task allocation but are restricted by ROS 1. Additionally, their dependence on PX4 limits their flexibility in adapting to other flight control systems.

Aerostack2 (Fernandez-Cortizas et al., 2024) presents an interesting ROS 2-based open-source solution that intends to overcome the shortcomings of many previous works (Pichierri et al., 2023; Real et al., 2020; Mohta et al., 2017). Its modularity by design allows the integration of different UAVs, whether they are off-the-shelf platforms or with an open architecture. Aerostack2 includes a rich software stack, from high-level mission control to basic robotic functions and sensor–actuator interface. However, although it is extremely powerful for low-level flight motion control and state estimation using sensor data, with its usability proven across many popular UAV platforms, including DJI and Crazyflie, Aerostack2 only integrates Behaviour Trees (Colledanchise and Ogren, 2018) for high-level planning. Its capability to increase autonomy in the coordination of multiple UAVs is therefore limited.

Complementary to Aerostack2^10^, AUSPEX is designed for integrating multi-UAV, with a focus specifically on the high-level intelligence in the coordination of the aerial platforms in a SAR operation. To the best of our knowledge, AUSPEX is also the only multi-UAV integration framework with an interface to Unreal Engine^11^, which is leveraged for software-in-the-loop simulation.

### 2.2 AI methodologies in rescue missions

AI methodologies have become integral to rescue missions, particularly in enhancing the perception capabilities of unmanned systems, enabling them to detect and segment their surroundings with high precision. Among the most frequently used are You Only Look Once (YOLO) v8 (Varghese, 2024) for real-time object detection and SAM2 (Ravi et al., 2024) for precise segmentation to draw object boundaries, making them invaluable for tasks that require a fine-grained understanding of the surroundings. Additionally, vision transformers, for example, the vision transformer (ViT) model (Dosovitskiy et al., 2021), have introduced a shift in visual data processing by leveraging self-attention mechanisms for robust feature extraction and improved generalisation. The growing availability of these open-source models enhances the perception abilities of unmanned systems to navigate and operate effectively in GPS-denied and unstructured environments.

Although the most substantial use of AI methodologies is related to perception abilities, automating a holistic decision-making process is becoming increasingly important, particularly as modern systems incorporate a growing number of complex unmanned subsystems, and human operators must not be overwhelmed. In the following subsections, we present previous works that support the evolution of this aspect.

#### 2.2.1 AI in decision-making

The most general problem in decision-making consists of finding a path (or a sequence of actions) to a goal state, given the current state. Automated AI planning tools tackle exactly this class of problems. Whereas the developed AI planning tools have been disparate, recent efforts intend to unify the tools under a single framework: Unified Planning (UP) (Micheli et al., 2025), a versatile python library offering a high-level API to define planning problems programmatically and to call planning engines to solve them. Although UP, to a certain extent, allows more than offline planning by also integrating capabilities for anytime planning, replanning, and plan repair, the fundamental idea remains finding a path to a given goal state using the assumed knowledge of the external world within which the agent interacts. Some frameworks extend UP to embedded systems via ROS 2 (Sadanandam et al., 2023).

On the other hand, reinforcement learning (RL) is widely used for decision-making under partial observability. Instead of a path to the goal state, we obtain a policy intended to guide the agent to the next step that bears the highest probability of reaching the goal. Reinforcement learning has been used in many robotics applications, for example, Room2Room (Anderson et al., 2017), where an agent learns to navigate to the goal via visual information. Building on Markov decision process (MDP)-based decision-making approaches, Chanel et al. (2012) extended the concept to UAVs by introducing a POMDP-based framework that integrates planning for perception and perception-driven decision-making, thereby enhancing real-time target detection and recognition under uncertainty.

Coordinating multiple UAVs in SAR missions often requires an efficient routing strategy to ensure optimal coverage of search areas while minimising response time and resource consumption, which can be modelled as multi-vehicle routing problems (MVRPs). Various solvers tackle this class of problems using heuristics such as genetic algorithms (Ribeiro et al., 2022) or adaptive large neighbourhood search (ALNS) (Ropke and Pisinger, 2006; Wu et al., 2022). Sacramento et al. (2019) demonstrated the effectiveness of ALNS as a solver for large instances of MVRPs in UAV-assisted routing scenarios.

Another promising development is the incorporation of large language models (LLMs) into the planning process. This enables the formulation of mission requirements in natural language, which is a major advantage. A distinction can be made between pure LLM planners and LLM-supported hybrid planners (Ma et al., 2024). The majority of LLM-only methods focus on natural language prompts, which emulate dialogues by providing new instructions in textual forms and by generating textual responses (Mu et al., 2023), to be either parsed into parameters and actions or given directly in the code format to be executed (Singh et al., 2023; Vemprala et al., 2024). Other works in this regard learn interactions with LLMs to obtain more grounded task models (Huang et al., 2023), enabling the symbolic representation of sub-goal plans (Zheng et al., 2022), the computation of smooth plan trajectories by combining LLM-generated and hand-sketched trajectories (Zu et al., 2024), or the pre-training of robots on specific tasks (Driess et al., 2023). Planning frameworks such as SayCan (Ahn et al., 2022) and SayCanPay (Hazra et al., 2024) are aligned with the position paper by Kambhampati et al. (2024), which advocates for LLM-modulo planning frameworks. These works use LLMs to suggest potential candidate actions; the suggestions are validated through grounding to determine whether the actions are feasible in the environment in which the robot operates and are subsequently executed if the feasibility is confirmed. Say’n’Fly (Döschl and Kiam, 2025) extends these concepts by developing an LLM-modulo online planning framework for UAVs in SAR missions. An LLM is leveraged to exclude lowly feasible actions, resulting in a smaller planning problem (by reducing the number of actions), whereas online heuristic search is exploited to decide for the next action with the lowest uncertainty in future rewards.

Selected AI tools for decision-making are integrated into AUSPEX as planning capabilities that are interchangeable, depending on the SAR scenarios.

#### 2.2.2 Knowledge representation and knowledge base

Effective planning in robotic systems requires a representation of the world and its current state. Classical planners rely on symbolic planning languages like PDDL (Ghallab et al., 1998; Fox and Long, 2003), which mostly only consider static knowledge. It lacks a way of taking dynamic knowledge, such as current sensor data, into account.

KnowRob (Tenorth and Beetz, 2017) suggests adhering to the syntax of the data structures of a robot’s control program while developing a knowledge base. KnowRob’s successor, KnowRob2 (Beetz et al., 2018), can be used as a query-answering system in the domain of complex robot manipulation tasks. Despite using the logic-based query language, the knowledge-processing framework has a hybrid structure consisting of multiple ontologies, along with logical expressions that are constructed on demand from episodic memories and low-level motion data; therefore, this enables tight coupling between low-level motion planning and high-level task planning. The AUSPEX framework focuses mainly on high-level tasks, abstracting the underlying motion parameters.

Galindo et al. (2008) demonstrated how to enhance (metric and topological) map information with semantic knowledge representation, which is useful for task planning in robotics. They split the knowledge into S(patial)- and T(erminological)-Box; the first contains spatial knowledge about the environment (e.g., coordinates of detected objects), whereas the second stores general concepts such as relationships between classes of objects. Other approaches utilise LLMs as they internalise rich commonsense knowledge through the huge amount of training data (Huang et al., 2022) or use a single knowledge graph constructed from encoding semantics and instance knowledge for UAV mission planning (Duan et al., 2024). This knowledge contains different categories: scenario (environmental data), resource (power), task (image processing), and performance (maximum flight speed) knowledge, and is stored in triples (drone1, speed-is, and 12).

## 3 Problem description of an SAR mission

In a typical SAR mission, UAV operators receive high-level mission goals from the central command and control unit, for example, locating an object or person (of given characteristics) in a search area, scanning, or mapping an area. The mission goals are expressed mostly in natural language and intermittently with numerical information.

In general, in every use case, the definition of parameters related to the available resources and the underlying mission is necessary. The UAVs available as resources for the underlying mission are represented by the set $U=\left\{u_{1},\ldots ,u_{M}\right\}$ , with each UAV, $u_{m}$ , possessing a set of skills^12^ $s_{u_{m}}={\left\{s_{1},\ldots ,s_{S}\right\}}_{u_{m}}$ and $S_{u}=\left\{s_{u_{1}},\ldots ,s_{u_{M}}\right\}$ , whereas $p_{m}$ describes the state of the UAV $u_{m}$ . Depending on the mission scenario, a set of (numerical and propositional) goal conditions $G=\left\{g_{1},\ldots ,g_{K}\right\}$ must be defined. Typical goals in SAR scenarios involve monitoring an area, thereby locating missing persons, delivering first aid, and establishing communication with survivors. The mission is considered accomplished when the goal conditions are satisfied. On the other hand, a set of constraints $C=\left\{c_{1},\ldots ,c_{J}\right\}$ must be defined, for example, response time or efficient energy consumption in emergency scenarios. For this, we leverage the definition of global constraints given by Scala et al. (2016), that is, a global constraint is a Boolean formula obtained from (numerical and propositional) conditions that must stand true at all times. At each time instant t , the current world state S(t) is known (e.g., map information, remaining resources, and detecting objects and their location). The solution to the coordination problem P is m sequences of actions, that is, plans, with each sequence of actions $\pi \left(u_{m}\right)={<a_{1},\ldots ,a_{N}>}_{u_{m}}$ being the plan for UAV $u_{m}$ .

Based on this general problem formulation, specific objective or cost functions can be modelled but are dependent on the use case and the considered planning problem class. Therefore, with additional definitions, we can enable the framework to adapt its planning strategy to mission-specific requirements, such as minimising response time or battery consumption or maximising coverage.

## 4 AUSPEX: system architecture

In this section, we describe AUSPEX, a multi-UAV open-source software framework designed with a modular architecture to which different planning capabilities are integrated for solving coordination problems. AUSPEX is well suited to solve SAR problems but is not limited to them as it can be applied to a range of other mission scenarios.

**FIGURE 1:**
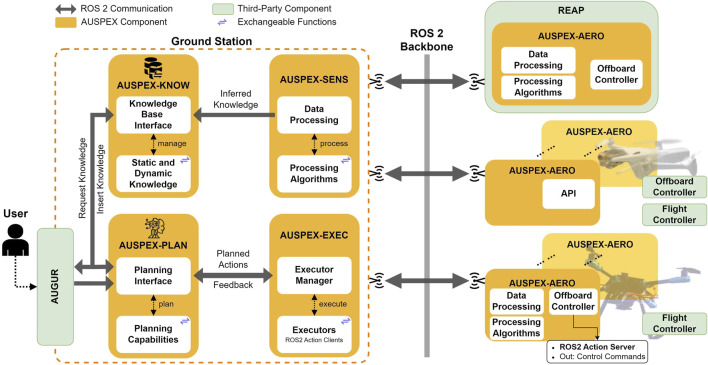
The AUSPEX framework contains five modules (i.e., AUSPEX-PLAN, AUSPEX-KNOW, AUSPEX-SENS, AUSPEX-EXEC, and AUSPEX-AERO) interconnected by the ROS 2-based communication backbone. Interchangeable functions within each module ensure that different algorithms or logics can be applied, depending on the class of mission.

At its core, the framework is composed of five key elements (see Figure 1):

1. AUSPEX-KNOW: a knowledge base that consolidates processed information on the world state S(t) up to the time instant t from the observations.
2. AUSPEX-SENS: a sensor data-processing module that generates new information from the observed data at time t to update the world state S(t) stored in AUSPEX-KNOW.
3. AUSPEX-PLAN: a module with planning capabilities meant for generating a coordination plan π based on S(t) to solve the given mission, that is, to reach the set of goal conditions G while ensuring that the plan trajectory satisfies the constraint C .
4. AUSPEX-EXEC: a module built to execute the plan π and monitor the execution.
5. AUSPEX-AERO: a module to be deployed onboard on the UAV^13^ to include real-time software stacks such as the offboard controller and sensor data-processing capabilities.

The AUSPEX framework uses ROS 2 (Macenski et al., 2022) as the communication backbone, thereby allowing inter-module communication through customisable ROS ∼ 2 messages. To communicate a computed plan to the offboard controller^14^ of the UAVs, whether these are real or simulated UAV platforms, the AUSPEX-EXEC uses the ROS 2 “Action Server and Action Client” principle. The UAVs, in turn, report their current state back to the system during the execution, enabling the AUSPEX-KNOW to acquire updated observation and telemetry data, whereas AUSPEX-EXEC continuously monitors the plan feasibility. Additionally, a graphical user interface (GUI) (see AUGUR in Figure 1) acts as a peripheral module, primarily displaying potential plans and the real-time operational picture of the UAVs while also enabling UAV operators to define mission goals.

AUSPEX is modular by design, that is, each module has its functionality and is composed of an interface designed to call interchangeable functions (see exchangeable functions in Figure 1). To emphasise the modularity of AUSPEX within its implementation, the modules are organised in different GitHub repositories. A central repository, AUSPEX, serves as the unified entry point to the system, providing hardware and software setup instructions, a quick start guide, a system overview, default settings, and links to each submodule of AUSPEX. AUSPEX is available as an open-source framework^15^. The following subsections provide more insights into the individual modules of AUSPEX.

### 4.1 AUSPEX-PLAN: the planning module

The planning module is a core component of AUSPEX^16^. This module is developed for finding a coordination plan for the mission described in Section 3, that is, finding an action sequence $\pi \left(u_{m}\right)$ for each UAV $u_{m}$ of a given team to achieve the goal conditions in G while acting under the constraints in C .

AUSPEX-PLAN is divided into two parts: planning interface and planning capabilities (see AUSPEX-PLAN in Figure 1). The three main functions of the planning interface are as follows:

1. to query AUSPEX-KNOW for parameters essential to define the planning problem, such as state parameters (e.g., available resources, initial positions of the UAVs, and geospatial information), constraints, and goal conditions;
2. to query AUSPEX-KNOW for the desired planning capability^17^ and call it upon planning request;
3. *to communicate the plan(s) to AUSPEX-EXEC* which, in addition to tasking the UAVs to perform the actions, also monitors the plan execution and triggers a replanning whenever necessary.

**FIGURE 2:**
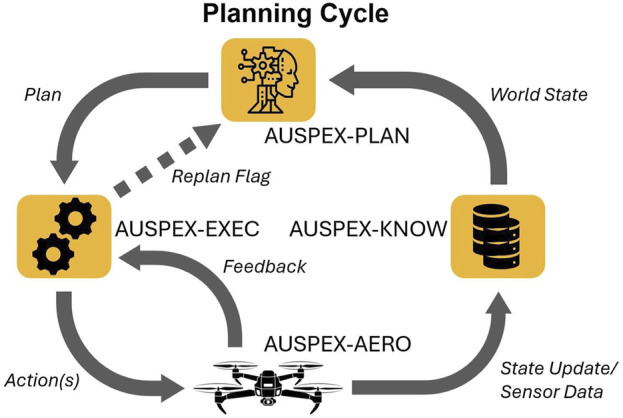
The planning cycle: the iterative process of planning, execution, and perception.

It is noteworthy that in this work, we consider a solution $\pi \left(u_{m}\right)$ to be a coordination plan consisting of sequences of actions, with each sequence associated to one UAV agent $u_{m}$ . The plan is encoded as a ROS 2 message Plan.action^18^. The schematic diagram of a planning cycle is shown in Figure 2.

By querying AUSPEX-KNOW, the planner gets the world state known at planning time and forwards the computed plans π to AUSPEX-EXEC, which communicates the actions $\pi \left(u_{m}\right)$ to the associated UAVs $u_{m}$ to be executed. The UAVs notify AUSPEX-EXEC about the status of current actions by sending feedback and result messages directly back to the executor. Additionally, the UAVs publish their current perceptions and drone states, containing, among others, position and state, to AUSPEX-KNOW, where the world state is continuously updated.

The second part of AUSPEX-PLAN serves as a container of planning capabilities. For the time being, four classes of planning capabilities are integrated into AUSPEX. These are as follows:

1. the automated planning capability, by integrating UP (Micheliet al., 2025), an ensemble of domain-independent planners,
2. heuristic-based multi-vehicle routing algorithms, by integrating ALNS from Wouda and Lan (2023) ^19^,
3. the LLM module planning framework, by integrating Say’n’Fly (Döschl and Kiam, 2025),
4. a pattern planner for computing simple flight trajectories to scan an area.

The planning capabilities are interchangeable, and more can be integrated. For this purpose, we also provide a mock planner, which is a skeleton planner for testing and prototyping new implementations and integration of planners. The planner for a mission scenario is currently selected by inserting a tuple consisting of the team_id and planner_id into AUSPEX-KNOW.

In the following sections, more details on the different planning capability classes will be provided.

#### 4.1.1 Unified planning: a library of AI planning engines

UP is a library powered by a collection of AI planners, with a unified interface in Python allowing either the definition of planning problems using standard domain-independent problem definition languages, such as Planning Domain Definition Language (PDDL) and Action Notation Modelling Language (ANML), or programmatically via a Python interface (Micheli et al., 2025). UP provides planning engines for classical, temporal, numeric, and hierarchical task network (HTN) planning problems, covering problem domains ranging from block world (Micheli et al., 2025) to real-world domains for UAVs (Kiam et al., 2020). Examples of planning engines include Fast Downward (Helmert, 2006) and Tamer (Valentini et al., 2020))^20^

To integrate UP into AUSPEX, we use UP4ROS2^21^, which is a library designed to interface UP functionalities with ROS ∼ 2 services. With the integrated OneShotPlanner client from UP4ROS2, a plan solution to the defined problem will be computed. The OneShotPlanner calls the respective plan function in UP, which automatically selects a planning engine, for example, Fast Downward for most simple problems. There are two ways to model the planning problem: the conventional way consists of pre-parsing the problem into a domain and problem instance^22^ in either PDDL^23^ or ANML. A more sophisticated way consists of encoding the problem programmatically through the UP interface, with an action space essential to model the problem domain and parameters to instantiate the problem, such as goals, constraints, and initial state, queried from AUSPEX-KNOW. The computed plan solutions are sequences of actions, with each sequence associated with an UAV, and are converted into the Plan.action ROS 2-message format standardised by UP4ROS2 to be returned to the planning interface. Our UP interface supports single UAV planning; however, multiple instances can be created to enable multi-UAV planning.

#### 4.1.2 Adaptive large neighbourhood search as a planning capability

Evolutionary algorithms have been used widely as schedulers in swarm technologies involving multiple agents. The problem described in Section 3 can be treated as a multiple-vehicle routing problem if one or more optimisation criteria are provided and multiple UAV agents are involved. We integrated ALNS (Wouda and Lan, 2023) as a solver in AUSPEX. We extended the AlphaUCB selector in the original implementation from Wouda and Lan (2023) by introducing an additional variable, β , which weighs the computation time of each repair or destroy operator. This modification provides the ability to prioritise response time over optimality, which is relevant for SAR operations, as the computation time is often a critical factor.

Similar to the use of UP, the planner interface queries AUSPEX-KNOW for parameters to model the MVRP, including optimisation criteria (i.e., total travelled distance, total mission duration, and number of deployed vehicles) and the timeout duration. Each obtained route for an UAV will be converted into the Plan.action ROS 2 message format.

#### 4.1.3 Say’n’Fly as an LLM-modulo planning capability

Owing to the ability to understand missions formulated in natural language and context that cannot be predefined formally, LLMs have been leveraged for decision-making (Huang et al., 2022; Zu et al., 2024). In order to avoid “wrong” plans, Kambhampati et al. (2024) proposed to resort to LLM-modulo frameworks when leveraging LLMs to plan. Aligned with this, Say’n’Fly is an LLM-modulo framework for online planning; it discards irrelevant or lowly feasible actions based on domain-specific knowledge to reduce the size of the planning problem while leveraging online heuristic search to reduce uncertainty (Döschl and Kiam, 2025). The performance of Say’n’Fly was assessed in the simulation environment REAP (Kraus et al., 2023) by evaluating the goal completion of multiple runs. However, if the natural language processing (NLP) included in Say’n’Fly cannot detect a high-level task due to ambiguous instructions, the input will be rejected. Compared to conventional planners, Say’n’Fly provides a more flexible interface by leveraging the commonsense knowledge of LLMs, which enables a more effective adaption to mission contexts in the open world.

Say’n’Fly queries the knowledge base for the mission description, which is a string parameter. Once the best possible action is computed, it will be parsed into the Plan.action format. It should be noted that here, only a single action is contained in the ROS 2 message. Although Say’n’Fly is designed for a single UAV, it allows UAV operators to quickly command single UAV in natural language without manual entries of mission parameters.

#### 4.1.4 Pattern planner

Often in SAR operations, UAVs are tasked to scan an area (and forward the video stream subsequently) in a given pattern. AUSPEX, therefore, integrates a pattern planner, which includes, but is not limited to, the computation of a lawnmower pattern based on the flight dynamics of the UAV platform and the field of view of the camera payload. Currently, the pattern planner computes a search area for single UAV, but multiple search areas can be defined to support multiple UAV.

The planning interface queries AUSPEX-KNOW for the polygon defining the mission area and converts the computed scanning path into actions to be encoded in Plan.action.

### 4.2 AUSPEX-EXEC: the execution module

The main function of AUSPEX-EXEC (see Figure 1) is to task the UAVs in real time according to the computed plans that are communicated via ROS 2 as Plan.action messages and to handle the feedback from the UAVs. The module is composed of an execution manager and executors. The execution manager creates one executor node for each connected UAV and spins it in a separate thread. The executor nodes use a ROS 2 action client to send the actions to the offboard controllers of the connected UAVs. The number of actions sent to the UAVs can be customised according to the type of platform and the level of autonomy the UAV operator would like to assign to the unmanned platforms. Based on the feedback from the UAVs, in case of a cancelled^24^ or aborted^25^ action, the AUSPEX-EXEC can trigger a “replan” and send this command back to AUSPEX-PLAN.

It is noteworthy that each executor is aware of the plans and its associated UAV agent, allowing it to control the execution of the actions and forward feedback to the planning module in case of an unexpected behaviour during execution that requires the planner to be involved (by replanning). In AUSPEX, the execution module is decoupled from the planning module. Furthermore, the execution module is also decoupled from the execution platforms. This enables AUSPEX adopting different execution logics for each UAV without altering the planning module while retaining more compatibility with various offboard controllers of off-the-shelf UAV platforms.

### 4.3 AUSPEX-AERO: the airborne module

In SAR operations, deploying heterogeneous UAVs is essential due to their varied mission capabilities (deriving from the different mission payloads) and computational capacity; for example, some are meant for in-flight object detection, and some play the role of a communication relay node. Moreover, in practice, having heterogeneous UAVs in the fleet is circumstantial as hardware is often acquired at different times, depending on available financial resources and on the hardware quality–price ratio at the time of acquisition. Ensuring interoperability of different UAVs, ranging from off-the-shelf platforms to platforms with an open architecture, can be challenging as the communication protocols, hardware limitations, and software compatibility with the onboard computational unit are all platform-dependent. AUSPEX-AERO^26^ is a module to be deployed onboard on the UAV, using companion computers such as a Raspberry Pi or an NVIDIA Jetson board. We developed an offboard controller included in AUSPEX-AERO.

In the following section, we provide details on the offboard control component of AUSPEX-AERO. It is noteworthy, however, that AUSPEX-AERO can also include sensor data processing components that are contained in AUSPEX-SENS if the onboard computational capacity supports them.

#### 4.3.1 Offboard controller design

**FIGURE 3:**
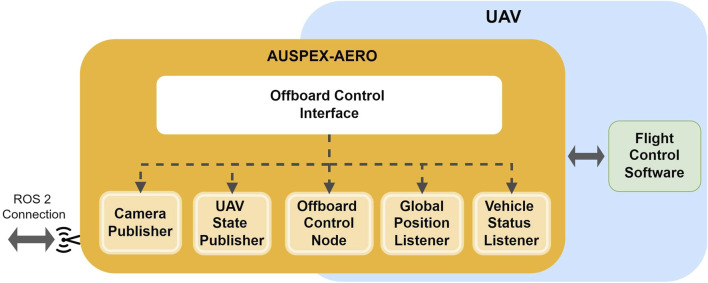
Composition of the offboard controller software stack: the components composing the offboard controller, including the control node, data publishers, and listeners, which interface with the flight control software.

The offboard controller in AUSPEX-AERO acts as the action server, receiving actions from AUSPEX-EXEC. It is in practice a ROS ∼ 2 node stack, as illustrated in Figure 3. The offboard control interface instantiates five nodes:

1. Camera Publisher starts the connection to a camera and launches the publisher with a fixed frames per second parameter.
2. UAV State Publisher takes the name of an UAV, its current position, orientation, and status, as well as the camera rotation, and wraps them into a message and publishes it frequently.
3. The Global Position Listener is a collection of subscribers connected to the flight control interface to obtain telemetry data from the UAV platform.
4. The Vehicle Status Listener, similar to the Global Position Listener, is also a collection of subscribers connected to the flight controller interface to collect status information managed by the flight controller.
5. The Offboard Control Node is the main node responsible for handling the other nodes by prescribing the intra-process communication logic while also computing the control commands for the planned actions received from AUSPEX-EXEC, including pausing and cancelling ongoing actions.

It should be noted that nodes of the offboard controller are written in C++ to take advantage of the shorter computation time.

**FIGURE 4:**
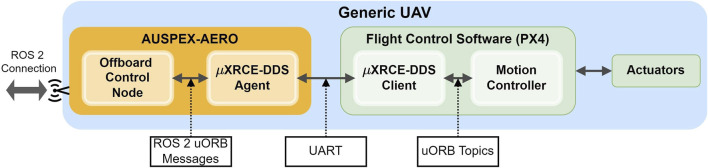
Communication interface between the offboard controller component in AUSPEX-AERO with a generic UAV running on a PX4 flight control.

The offboard control unit is platform-dependent. The integration of several examples of UAV platforms is provided. We outline here specifically the integration with a generic platform using PX4 (Meier et al., 2015) as the autopilot software. As illustrated in Figure 4, the interfacing with a PX4 autopilot is performed using an μ XRCE-DDS agent^27^. The Offboard Control Node translates actions received from AUSPEX-EXEC into control commands encoded into an ROS 2 message tailored for the specific UAV platform. The message is published as an ROS 2 topic and made available by the μ XRCE-DDS Agent for subscription by the μ XRCE-DDS Client. Subsequently, the uXRCE-DDS client translates ROS 2 messages into uORB topics, which are processed by the flight controller.

**FIGURE 5:**
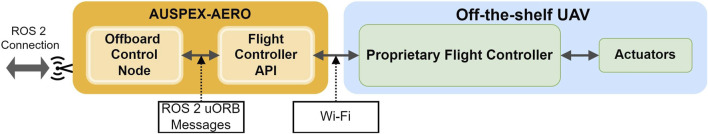
Example integration of a UAV into AUSPEX with a proprietary non-open-source flight controller.

In addition to a generic UAV platform using a PX4 autopilot, we also integrated an off-the-shelf UAV with a proprietary non-open-source flight controller. Figure 5 depicts the slightly altered architecture for the integration of a Parrot Anafi AI. The Olympe SDK serves as the interface for the Offboard Control Node. Notably, the connection between this interface and the flight controller is wireless. Due to the closed architecture of such an off-the-shelf UAV platform, AUSPEX-AERO is hosted by hardware external to the UAV platform^28^.

### 4.4 AUSPEX-SENS: the sensor data-processing module

AUSPEX-SENS is a module with the main functionality of extracting information from a continuous data stream obtained by sensors mounted onboard of the integrated UAVs. Object detection is an indispensable image processing tool in highly automated SAR operations. The object detection model, YOLOv8 from Varghese (2024), is capable of multi-object recognition and is integrated into AUSPEX-SENS. In view of increasing its usability for processing images from aerial perspectives, we specifically leverage a version of YOLO pretrained on the dataset COCO (Lin et al., 2014) and fine-tune it using a modified version of the VisDrone (Zhu et al., 2021) dataset. The fine-tuning is domain-specific, involving class reduction to streamline detection tasks and optimise performance specifically for SAR operations. In addition to YOLOv8, AUSPEX-SENS can be extended to include other tools such as ZegClip (Zhou Z. et al., 2023), a zero-shot vision-language model suitable for scenarios requiring robust generalisation across diverse object classes without extensive retraining, or SAM2 (Ravi et al., 2024), a cutting-edge model that excels in precise segmentation and is adaptable to complex use cases involving intricate object boundaries.

**FIGURE 6:**
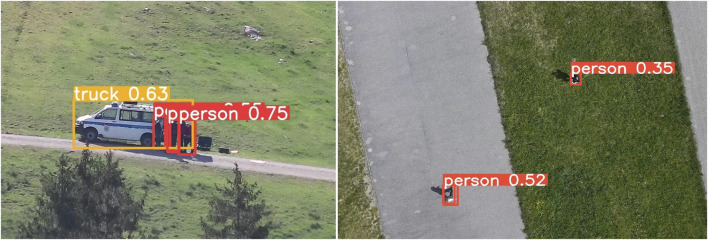
Images captured from different aerial perspectives: the left shows an oblique view and the right shows a nadir view.

Figure 6 showcases the results of the object detection applied to real images captured by an UAV. The detection performance is generally robust, with challenges arising in nadir images, as in the right image in Figure 6, mainly because this perspective is often not a part of the commonly used training datasets.

### 4.5 AUSPEX-KNOW: the knowledge base

AUSPEX-KNOW^29^ serves as a centralised source of knowledge for any module of AUSPEX, storing mission parameters defined in Section 3, that is, U , C , G , $S_{u}$ , and S(t) . Each component of AUSPEX can query, insert, update, or delete knowledge through a unified interface via ROS 2 services. The module AUSPEX-KNOW is separated from the consuming components, which increases the adaptability of AUSPEX, as the knowledge base can be updated, restructured, or mirrored independently.

**FIGURE 7:**
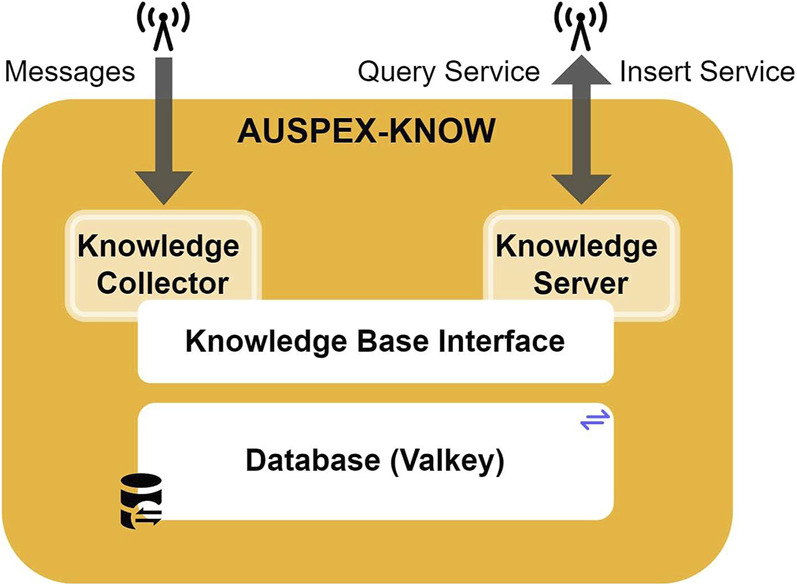
Components of AUSPEX-KNOW.

The knowledge collector and the knowledge server are the two interfaces provided by the knowledge base interface in AUSPEX-KNOW for managing the data inside the database, as shown in Figure 7. By using the knowledge server, data can be inserted (InsertKnowledge.srv) or updated (UpdateKnowledge.srv) directly via ROS 2 services^30^. The knowledge collector, on the other hand, listens to relevant messages (e.g., ROS 2 messages containing UAV state), position (i.e., the PlatformState.msg), or information on detected objects (i.e., the ObjectKnowledge.msg) and inserts either relevant messages automatically into the database, formatted as JSON, or the relevant data fields of a previously stored message are updated, given that the message can be associated with a unique key, that is, the platform_id. A PlatformState.msg, for example, entails the platform ID, the team ID, the GPS position, the NED position, the battery state, and the current platform status.

Relevant information includes the platform positions, platform states, and information extracted from processed sensor data, which are considered *dynamic* knowledge, as these require regular updates during plan execution. Additionally, platform capabilities, resources, mission requirements, and operational map data are stored without regular updates as they remain true during plan execution. In AUSPEX-KNOW, the knowledge is represented in a frame-based manner and is organised in collections, in which each collection contains frames for entities. Technically, a frame is implemented as a JSON dictionary, containing attribute–value pairs. Queries can be formulated in JSONPath-based query language. Valkey^31^, which is an open-source variant of Redis^32^, is set up as the backbone of the database in AUSPEX-KNOW. Valkey offers a module to store these JSON data, allows (quasi) real-time caching, and meets the requirement of flexibility through its schema-less nature.

### 4.6 Connecting to an external mission control interface—AUGUR

Regardless of the level of automation, the operation of UAVs in SAR operations requires a human in or on the loop. For mission control and supervision, AUSPEX can be connected to AUGUR^33^, which is a GUI intended for human–machine interaction^34^. The GUI is developed using Flutter, a versatile framework that supports cross-platform deployment, from desktop computers to mobile devices. Using the rosdart library, AUGUR establishes a connection with AUSPEX’s ROS 2 ecosystem and with AUSPEX-KNOW, allowing it, for example, to query and display real-time information.

### 4.7 Connecting to an external simulation environment—REAP

**FIGURE 8:**
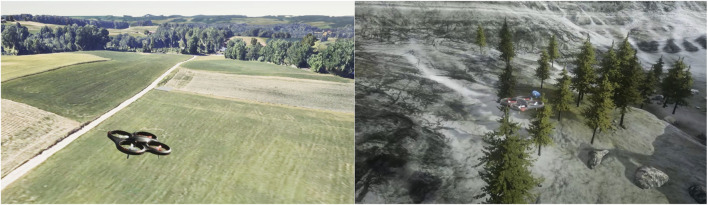
Simulated UAVs connected to AUSPEX in different environments of Unreal Engine.

AUSPEX can be connected to REAP^35^, which is a realistic simulation framework for aerial applications. REAP is based on Unreal Engine 5.2^36^ and uses AirSim (Shah et al., 2017) for simulating the flight dynamics of UAVs, various weather conditions, and cameras (i.e., electro-optical and infrared imagery) mounted on the UAVs (Kraus et al., 2023). Through the use of the Cesium plug-in and Google photorealistic tileset, AUSPEX can be deployed on simulated UAVs spawned in (simulated) locations. Figure 8 depicts a flight above a green flatland in Bavaria, Germany.

## 5 Example SAR use cases of AUSPEX

In this section, we demonstrate the use of the AUSPEX in a few SAR-related example use cases. The configuration of these use cases can be found in the AUSPEX repository^37^. The use cases are either tested using a simulated UAV platform in the REAP simulation environment or a with Holybro X500 v2 as the UAV platform, equipped with a Pixhawk 6C flight controller, a Raspberry Pi 5 as a companion computer, and a Wi-Fi dongle with a connection to the mobile network. This allows us to connect the UAV with a VPN to the ROS ∼ 2 network of the AUSPEX ground segment.

### 5.1 Use case 1: a search mission

The goal of the use case is to locate a designated object at several locations (i.e., points of interest). For this, UP is used, with the goal conditions having all the points of interest visited by the UAV. We use the OneShotPlanner function from UP to call the Fast Downward planner^38^ (Helmert, 2006) to compute a plan. The expected plan quality can be configured in UP using the OneShotPlanner, and it is set to be optimal with respect to the number of actions as actions are assumed to have unit cost. The setup for testing this use case is shown in Table 1.

**TABLE 1:** The set of parameters and modules used in a search mission.

Flight controller	Autopilot interface	AUSPEX-PLAN	AUSPEX-SENS	Goal conditions G
Pixhawk 6C (Holybro X500)	PX4	Unified planning (automated planning)	YOLOv8	All points of interest are visited

Plans generated by UP achieve a hundred percent execution success rate as the goal of visiting all waypoints in this use case is simple without any further constraints and only fails in case of UAV malfunctions. The planning time of UP is highly dependent on the complexity of the defined constraints on actions and goals. In this scenario, the computation time is approximately 1 s.

#### 5.1.1 Test outcome

Once the computed actions are confirmed, AUSPEX-PLAN forwards them to AUSPEX-EXEC. Listing 1 shows the ensemble of multiple actions as ActionInstance.msg objects from up_msgs and their respective parameters as part of a Plan.msg object.

Listing 1 A Plan.msg for the UAV with the attributed identity vhcl0.

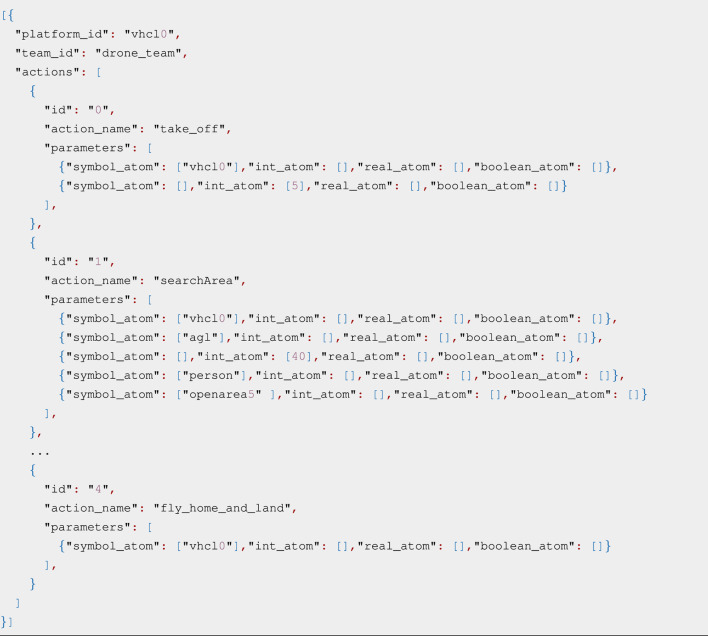

For each execution platform of the selected team (team_id), a Plan.msg object is generated with the respective keys platform_id and team_id (i.e., identity of the team to which the UAV belongs). The key actions entail a list of all ActionInstance.msg objects associated with the respective platform. An action message is defined by a unique identifier id, its name in natural language, and several parameters in the up_msgs/Atom.msg format, parameterising the action. Atoms can be used to define various parameters, such as waypoints by their name or GPS position, goal heights in whole numbers, search areas, search objects, and execution platforms.

### 5.2 Use case 2: reconnaissance mission

Reconnaissance is essential to survey an area using aerial imagery, especially before penetrating a hazardous operational area with manned units.

**FIGURE 9:**
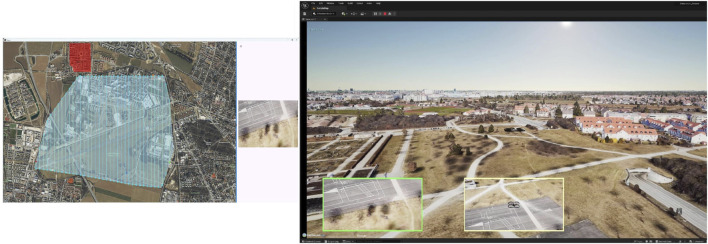
The window to the right displays REAP with the simulated UAV and the photorealistic map. In the simulation, two camera windows can be observed: one is the camera view of the UAV, whereas the other is the third-person camera view. The left represents the GUI AUGUR, displaying the planned lawnmower pattern to scan the area, whereas the smaller window to the right is the camera view.

Table 2 shows the setup for testing AUSPEX on this use case. The use case was performed within the simulation environment REAP, using AirSim to simulate the UAV flight physics and PX4 as the autopilot. As the input for the mission definition, one or more search areas are defined and sent to AUSPEX-KNOW. The search area is displayed on the GUI of AUGUR on the left side of Figure 9. The search area is defined in a SearchArea.msg file.

**TABLE 2:** The set of parameters and modules used in a reconnaissance mission.

Flight controller	Autopilot interface	AUSPEX-PLAN	AUSPEX-SENS	Goal condition G
REAP + AirSim	PX4	Pattern planner	YOLOv8	Camera footprint covers the search area

As the goal of the pattern planner is to cover a search area, the success rate can be measured accordingly. In practice, the executed patterns achieve approximately 80– 90 % coverage on average. The planning time depends on the number of waypoints the planner has to generate, which increases with the size of the search area and decreases with the UAV field of view.

#### 5.2.1 Test outcome

The pattern planner uses a lawnmower pattern to compute an optimal path with respect to area coverage, taking into account the field of view and desired operation altitude of the UAV. The computed waypoints are shown in Figure 9 in the left GUI window. These waypoints are translated into a list of actions and are sent to the respective UAV, which executes the fly actions. The object detection remains the same, and for each detected object, a message file is generated (see Listing 2), published, and inserted into AUSPEX-KNOW by its collector function.

Listing 2 An ObjectKnowledge.msg for one detected object.

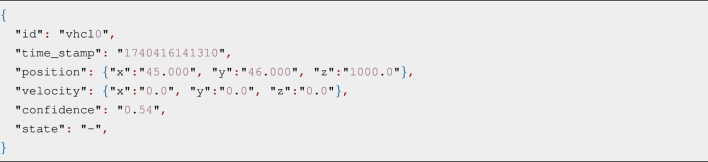

An exemplary ObjectKnowledge.msg object is shown in Listing 2, where the field id is the data source, together with a time stamp, the detection location, and confidence. With this approach, any object classes that the object detection model has not been explicitly trained on are ignored, suggesting that they cannot be detected or monitored during the mission. This limitation underscores the importance of selecting or training the detection model to include all relevant classes for the specific scenario.

### 5.3 Use case 3: dynamic mission control

Offline planning is not always feasible, especially in a partially observable environment. In this case, a UAV operator typically decides the next action of the unmanned vehicle based on real-time feedback. In current practice, the action command is formulated in natural language and executed by the UAV pilot and payload operator. In a highly automated planning and execution loop, the goal can be formulated in natural language, whereas AUSPEX searches for sequential actions and carries out the execution of these. The experimental setup of this use case is similar to the previous setup. The simulation environment with AirSim is used, together with AirSim’s simulated quadcopter. Here, we deploy the Say’n’Fly planning framework and consider a natural language input to define goal conditions, as illustrated in Table 3.

**TABLE 3:** The set of parameters and modules used in a dynamic search mission.

Flight controller	Autopilot interface	AUSPEX-PLAN	AUSPEX-SENS	Goal condition G
Simulated (REAP + AirSim)	PX4	Say’n’Fly	YOLOv8	Target detected (target description formulated in natural language)

Say’n’Fly computes non-optimal but satisfactory plans, when possible, due to the non-deterministic property of LLMs. The success rate is coupled to the object detection quality (i.e., currently ≈60 % ), and the computation time ( ≈3  s) is related to the evaluation of the LLM prompts or API requests. The natural language input contains the object(s) to be searched for and potential search sub-areas.

#### 5.3.1 Test outcome

In this example, the natural language input is set as follows: *Search for a person with a blue jacket. The person was last seen in open fields.* With this input, the Natural Language Understanding (NLU) tool yields person as the search target, blue as a supplementary attribute, and openareas as the designated search areas. These parameters are then processed by the Say’n’Fly planner to determine sequentially the next action following the feedback obtained from the observation data during execution. After each completed action, the planner receives the new UAV state, along with the current detections as the input, and determines the next action together with the natural language instruction.

### 5.4 Use case 4: multi-UAV coordination

SAR operations can take place over a wide area, in which case, a single vehicle is often insufficient due to limited flight duration, resulting in inadequate mission efficiency. Multiple UAVs can be a key solution. The deployment of multiple UAVs in an SAR operation consists mainly of assigning operational areas to each vehicle. This is analogous to an MVRP and can be solved using the ALNS solver in AUSPEX-PLAN. The respective parameters for this use case are given in Table 4.

**TABLE 4:** The set of parameters and modules used for multi-UAV coordination.

Flight controller	Autopilot interface	AUSPEX-PLAN	AUSPEX-SENS	Goal condition G
Simulated (REAP + AirSim)	PX4	ALNS	YOLOv8	14 Points of interest visited

In ALNS, several termination criteria can be defined, such as maximum time, maximum iterations, or other criteria that directly influence the resulting plan quality. However, in general, the plan quality is not guaranteed to be optimal. The computational cost (and thus the computational time) depends on the desired plan quality as higher plan quality can significantly increase the computation time. Running 1,000 iterations with ALNS can take up to 60 s. With two vehicles, a minimum of 500 iterations is typically required to ensure convergence to a locally optimal solution. In terms of scalability, the number of iterations required to reach a near-optimal solution increases with the number of vehicles. However, this can be mitigated by using pre-clustering algorithms (e.g., KMeans) to distribute the waypoints more effectively.

#### 5.4.1 Test outcome

**FIGURE 10:**
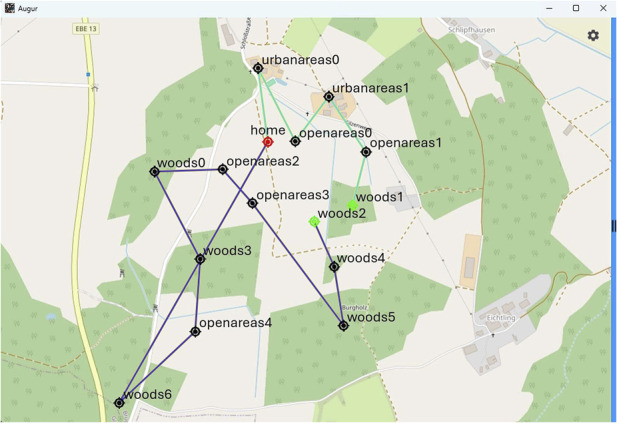
The generated waypoints for two vehicles, both starting/ending at the “home” position.

The solution with two vehicles and 14 points of interest is displayed in Figure 10. Two routes can be distinguished, each representing the route for an UAV.

The routes optimise (i.e., minimise) the total travel distance while being subject to the maximum flight duration of the platforms. Both UAVs start at the “home” position, and one covers the bottom left and the other covers the top right area. The waypoints are converted into one Plan.msg for each vehicle accordingly.

## 6 Comparison with existing frameworks

To provide an overview, a comparative analysis is shown in Table 5, outlining the distinctive features and advancements introduced by the AUSPEX framework. Several key dimensions of UAV frameworks are compared qualitatively to existing frameworks. Although some frameworks already offer robust solutions to specific use cases, AUSPEX demonstrates flexibility, scalability, and high-level planning capabilities across various use cases.

**TABLE 5:** Comparison of UAV frameworks across key features. (BT = behaviour trees).

Framework	Modularity	Open source	High-level planning	Heterogeneous UAV support	Mission control interface	Simulation integration	Execution architecture
Auterion^39^	Yes	No	Proprietary	Proprietary	Yes (App)	No	Centralised
Hivemind^40^	Proprietary	No	Proprietary	Proprietary	Yes	Limited	Decentralised
Aerostack2^41^	Plugin-based	Yes	Limited	Yes	Yes	Yes (Gazebo)	Centralised/distributed
CERLAB-UAV^42^	Limited	Yes	No	No	No	Yes	Centralised
MRTA E. A^43^	Limited	Yes	Partial (BT)	No	No	Yes	Centralised
AUSPEX	Fully modular	Yes	Yes	Yes	Yes (AUGUR)	Yes (REAP, UNREAL-based)	Centralised/distributed

In contrast to the CERLAB-UAV (Xu et al., 2023) framework and the MRTA execution architecture (Calvo et al., 2022), AUSPEX benefits from its *highly modular* design in deployment and operation. Being entirely *open-source*, AUSPEX encourages transparency and community collaboration, whereas Hivemind and Auterion are commercially available closed-source projects. AUSPEX-PLAN contains *several high-level planning and scheduling capabilities*, including UP, ALNS, and Say’n’Fly, and provides an interface for extending AUSPEX with more planners, offering more planning capabilities than the other approaches. Considering the design of AUSPEX-AERO for *heterogeneous UAVs*, AUSPEX supports integrating any UAV, including simulated ones, as long as a respective interface is available, where frameworks like Auterion support only UAVs equipped with their proprietary flight controller. As the mission planner or user interface, Aerostack2, CERLAB-UAV, and MRTA execution architecture utilise the open-source ground control station, QGroundcontrol^44^, whereas AUSPEX provides its own, *open-source GUI*, AUGUR, tailored specifically for AUSPEX, a unified and consistent interface to the flight controller, eliminating potential conflicts from parallel interfaces like QGroundControl. Leveraging ROS 2 as middleware allows a distributed deployment of the execution pipeline compared to approaches such as CERLAB-UAV or MRTA execution architecture, which are limited by ROS 1.

## 7 Conclusion

In this work, we introduced AUSPEX, a modular and open-source decision-making support system, as a scalable framework for integrating AI methods in coordinating UAVs. Building on a ROS 2-based communication backbone, the AUSPEX framework comprises five modules, namely, AUSPEX-PLAN for planning, AUSPEX-KNOW for knowledge storing and sharing, AUSPEX-SENS for sensor data processing, AUSPEX-EXEC for plan execution, and AUSPEX-AERO for onboard intelligence. AUSPEX can be integrated with the simulation frameworks REAP and AUGUR, the human–machine interface, thereby allowing software-in-the-loop validation and human-in-the-loop operations. In this paper, we demonstrated the use of AUSPEX in various SAR scenarios, including searching for a target object, surveying an area, coordinating multiple vehicles, and enabling dynamic mission control. Although AUSPEX-PLAN contains the intelligence to coordinate the UAVs, other modules are also essential to ensure the entirety of the system that is interconnected with the UAV agents acting in the real world and is capable of coordinating multiple agents with the same background knowledge.

The AUSPEX framework relies on open-source standards for transparency and also for future functional extensibility. AUSPEX uses ROS 2 to ensure modularity; that is, each module can be used as a stand-alone one and can also be mirrored for creating duplicates of them in a distributed multi-UAVs coordination system. In this work, we have also demonstrated the possibility of integrating heterogeneous UAV platforms into the AUSPEX ecosystem, which is advantageous for first-responder organisations involved in SAR operations as the system can adapt to different hardware requirements, where these are mission-dependent or logistically limited. Additionally, we demonstrated that AUSPEX can be connected to a realistic Unreal Engine-based simulation environment, facilitating realistic testing and software-in-the-loop validation.

### 7.1 Discussions and future work

Although the system demonstrates significant flexibility and adaptability, it has some limitations that need to be addressed. One limitation is the system’s reliance on a centralised knowledge-based architecture, which can become a bottleneck when handling a large number of UAVs or high-frequency data updates. This centralised approach may lead to delays in decision-making, or more precisely in the continuous monitoring of plan feasibility, particularly in scenarios involving multiple UAVs operating in real-time settings. Moreover, scalability is contingent on network performance as the system transmits significant overhead in real time due to the multicast communication architecture of ROS 2, including video streams and telemetry data. This dependency on robust and high-speed network infrastructure could pose challenges in bandwidth-limited or remote areas, an aspect that is not considered in this work.

Future efforts will focus on several key improvements to enhance the robustness and scalability of AUSPEX, especially in its deployment for hardware-in-the-loop flights. Optimising the network architecture to transmit only essential data and to include distributed computing capabilities will reduce overhead and improve efficiency, particularly for multi-UAV operations. A promising approach is to deploy the Fast DDS discovery server by eProsima^45^, which can reduce communication overhead by up to 90% (Macenski et al., 2022). Additionally, the system will be expanded to support other UAV pilot software, such as Ardupilot, and to integrate more diverse planning capabilities in AUSPEX-PLAN tailored to various mission types. Furthermore, an automatic planner selection step will be integrated in AUSPEX-PLAN to reduce the necessary manual input. These developments will ensure that the system continues to meet the demands of dynamic, real-world scenarios that are beyond SAR operations. To address the limitations of a centralised knowledge base, future efforts will focus on implementing a distributed knowledge base. Moreover, state-of-the-art knowledge representation and reasoning will be included in AUSPEX-KNOW, enabling the module with inference capabilities that can be essential when acting in the open world. An interesting enhancement is the deployment of a distributed Bayesian information fusion technique (Pavlin et al., 2010).

By providing a holistic, open-source, and modular multi-UAV decision-making support framework, AUSPEX represents a significant step forward in leveraging AI for aerial robotics deployed in emergency response. AUSPEX provides a base for future functional extensions and a framework ready to be deployed as a whole or only partially by first responders.

## Data Availability

The original contributions presented in the study are included in the article/Supplementary Material; further inquiries can be directed to the corresponding authors.

## Footnotes

1 Currently, the official German Recommendations for Common Regulations on the Use of Drones in Civil Protection (EGRED 2) (Bundesamt für Bevölkerungsschutz und Katastrophenhilfe, 2024) requires at least four operators per UAV in more complex scenarios.

2 Contrary to benchmarking use cases in research works, many standard procedures in civil applications exclude fully autonomous decision-making systems. Moreover, multi-UAV operations often require the aerial platforms to operate either at different altitudes or distinct operation areas. Therefore, increasing autonomy in the deployment of UAVs for SAR-operations often means the provision of an end-to-end decision-making support tool that eases the tasking of single UAVs while also helping to solve multi-vehicle routing problems.

3
https://auterion.com/

4
https://shield.ai/hivemind/

5 ROS 1 is reaching End-of-Life in mid-2025.

6 Aerostack2 only provides an interface to Gazebo (Koenig and Howard, 2004).

7 When deploying AUSPEX in simulation, scalability is mainly constrained by the performance of Unreal Engine and AirSim (Shah et al., 2017), while in real-world deployments, high-resolution image streams can saturate available bandwidth, making network capacity the primary limiting factor. The *scalability* of AUSPEX refers solely to that of the software stack and particularly on multiplying the modules to accommodate more UAVs.

8
https://auterion.com/

9
https://shield.ai/hivemind/

10 Given the modular ROS 2-based software architecture of both frameworks, software modules of Aerostack2 and AUSPEX are interchangeable. Both frameworks are concurrent, yet complementary.

11
https://www.unrealengine.com/

12 Skills of a UAV are also feasible actions of the platform.

13 In some cases, AUSPEX-AERO cannot be hosted onboard, for example, if the possibility of integrating a companion computer onto the UAV is excluded. In this case, AUSPEX-AERO can also be in the vicinity of and with a stable connection to the UAV.

14 The offboard controller stack is deployed on a companion computer, processes the given actions, and sends low-level commands to the flight controller.

15
https://github.com/UniBwM-IFS-AILab/AUSPEX

16 A dedicated repository for the Planner Module can be found here: https://github.com/UniBwM-IFS-AILab/AUSPEX-PLAN

17 The choice of a planning capability is stored in a configuration file.

18 The definitions of our messages can be found on GitHub in our AUSPEX-MSGS: https://github.com/UniBwM-IFS-AILab/AUSPEX-MSGS repository.

19 ALNS is in general known as a solver to scheduling problems, rather than a planner. We refer to ALNS as a class of planning capability by abuse of language.

20 For more details on the planning engines, see https://unified-planning.readthedocs.io/en/latest/engines/01_available_engines.html

21
https://github.com/aiplan4eu/UP4ROS2

22 Domain and problem instances are text scripts formalised using standardised problem definition languages such as PDDL and ANML.

23 For an example SAR PDDL setup: Domain and Problem file.

24 An action can only be cancelled manually by the UAV operator.

25 An action is aborted if it cannot be executed because its preconditions are not satisfied.

26
https://github.com/UniBwM-IFS-AILab/AUSPEX-AERO

27 Due to its real-time capabilities, the eProsima’s uXRCE-DDS is preferred to the standardised MAVLink as a middleware for onboard intra-process communication.

28 As the communication between the Flight Controller API and the onboard flight controller is wireless, AUSPEX-AERO can be hosted by a companion computer mounted onboard of an off-the-shelf UAV, as long as the additional peripheral computer does not alter the flight dynamics substantially.

29
https://github.com/UniBwM-IFS-AILab/AUSPEX-KNOW

30 The respective message collection can be found in AUSPEX-MSGS.

31
https://valkey.io/

32
https://redis.io/de/

33
https://github.com/UniBwM-IFS-AILab/AUGUR

34 AUGUR also comes with a Command Line Interface (CLI), that is mainly for back-end development purposes.

35
https://github.com/UniBwM-IFS-AILab/REAP

36
https://www.unrealengine.com/

37
https://github.com/UniBwM-IFS-AILab/AUSPEX/tree/main/utils/examples

38
https://github.com/aibasel/downward

39
https://auterion.com/

40
https://shield.ai/

41
https://github.com/aerostack2/aerostack2

42
https://github.com/Zhefan-Xu/CERLAB-UAV-Autonomy

43
https://github.com/grvcTeam/aerialcore_planning

44
https://qgroundcontrol.com/

45
https://fast-dds.docs.eprosima.com/
